# The relationship between social support and mental health in athletes: a systematic review and meta-analysis

**DOI:** 10.3389/fpsyg.2025.1642886

**Published:** 2025-09-03

**Authors:** Jianing Luo, Rui Du, Xiaolin Wang, Liang Luo

**Affiliations:** ^1^School of Physical Education, Shandong University, Jinan, China; ^2^Department of Physical Education, Liaoning Institute of Science and Engineering, Jinzhou, China; ^3^School of Physical Education, Ludong University, Yantai, China; ^4^School of Economics and Management, Wuhan Sports University, Wuhan, China

**Keywords:** social support, mental health, wellbeing, anxiety, depression, stress

## Abstract

**Introduction:**

Social support is widely recognized as a key determinant of athletes’ mental health; however, inconsistencies remain regarding the strength and source-specific effects of this relationship. This meta-analysis systematically quantifies the association between social support and mental health outcomes in athletes and examines variations by support source.

**Methods:**

A systematic search was conducted across multiple databases to identify relevant studies. Random-effects models were used to calculate pooled effect sizes expressed as correlation coefficients (*r*). Subgroup analyses compared the associations of family and friends’ support versus team-based support with mental health indicators.

**Results:**

Forty studies encompassing 14,462 athletes were included. Overall social support correlated positively with well-being (*r* = 0.31) and negatively with anxiety (*r* = −0.22), depression (*r* = −0.27), and stress (*r* = −0.25). Notably, support from family and friends showed a significantly stronger negative association with depressive symptoms than team-based support.

**Discussion:**

In conclusion, social support plays a vital role in enhancing athletes’ mental health, particularly through close interpersonal relationships. These findings underscore the importance of integrating diverse sources of social support in interventions aimed at improving psychological well-being in athletes.

## Introduction

It is widely recognized that regular participation in sport is associated with improved mental health and enhanced psychological well-being (Mahindru et al., 2023). However, while sport participation may serve as a protective factor, athletes are concurrently exposed to a constellation of stressors in their pursuit of competitive excellence (Daley et al., 2023). These include excessive training demands, dense competition schedules, intense media scrutiny, and the psychological burden of injury-related career uncertainty (Haugen, 2022). Such cumulative pressures have been consistently linked to elevated levels of depression and anxiety, threatening both performance and overall well-being. Recognizing these risks, recent research has increasingly focused on athletes’ mental health, emphasizing the prevalence and impact of psychological disorders across competitive levels and prompting growing interest in psychosocial resources, particularly social support, as potential buffers against psychological distress (Küttel and Larsen, 2020; Stevens et al., 2024).

Social support has long been recognized as a critical factor in mitigating the adverse psychological effects of stress and enhancing psychological resilience (Bedaso et al., 2021; Zhou and Cheng, 2022; Stevens et al., 2024). For athletes, who often operate in high-pressure environments characterized by intense physical training, performance expectations, limited recovery time, and public scrutiny, social support plays a particularly vital role in preserving mental health (Nuetzel, 2023; Simons and Bird, 2023). In addition to the physical and emotional challenges inherent in competitive sport, athletes may also experience prolonged time away from family, fear of underperformance, and conflicts between personal and athletic identities, all of which increase their reliance on external support (Stevens et al., 2024). Social support is typically provided by key figures in an athlete’s immediate environment, including family members, friends, coaches, and teammates. These individuals help shape both the perception and availability of support, which are essential in responding to psychological stressors. The stress-buffering model (Cohen and Wills, 1985) provides a widely accepted theoretical explanation for how social support influences mental health outcomes. This model suggests that social support reduces the negative psychological impact of stress by facilitating emotional regulation, fostering adaptive coping strategies, and diminishing the perceived severity of stressful experiences. Within the context of sport, this framework has been used to explain how supportive interpersonal relationships may protect athletes from anxiety, emotional exhaustion, and other mental health challenges (Delfin et al., 2024; Hartley et al., 2023). Complementing this view, the dual continuum model of mental health (Keyes, 2002) emphasizes that mental health is not merely the absence of psychopathology, but also the presence of positive psychological functioning. From this perspective, social support not only helps reduce negative symptoms such as anxiety and depression but also contributes to enhanced psychological flourishing and well-being.

Although numerous studies have underscored the potential positive effects of social support on athletes’ mental health, including reduced anxiety and depression as well as enhanced well-being, the empirical findings remain inconsistent. Some studies report significant associations between higher levels of social support and better mental health outcomes (DeFreese and Smith, 2014; Hagiwara et al., 2017, 2021; Simons and Bird, 2023), whereas others find weak or non-significant associations (Price and Weiss, 2000). This inconsistency may stem from differences in how social support is conceptualized and measured across studies, particularly in relation to the sources of support (e.g., family, coaches, teammates). The role of these sources may vary, with some providing more meaningful psychological benefits than others depending on the context.

Furthermore, individual factors such as gender and athlete level can moderate the effectiveness of social support. Female athletes may face unique psychological challenges, such as societal gender expectations and role conflicts, which could shape their use of social support (Cnen et al., 2021). On the other hand, male athletes may be less likely to seek support due to cultural norms around emotional expression. Additionally, elite athletes often experience higher performance pressures and career uncertainties, which may lead to more specialized support needs compared to recreational athletes (Kuok et al., 2021; Reardon, 2021). However, further research is required to better understand how these factors interact with social support and affect mental health outcomes.

Despite existing studies, no comprehensive meta-analysis has yet quantified the relationship between social support and mental health in athletes or explored how individual factors such as gender and athlete level, as well as different sources of support, influence this relationship. This study aims to conduct a systematic review and meta-analysis to assess the strength and direction of this association and examine potential variations based on support sources. By synthesizing existing evidence, this study seeks to clarify inconsistencies in the current literature and provide a robust empirical foundation for future intervention strategies.

## Methods

### Search strategy and study selection

**FIGURE 1:**
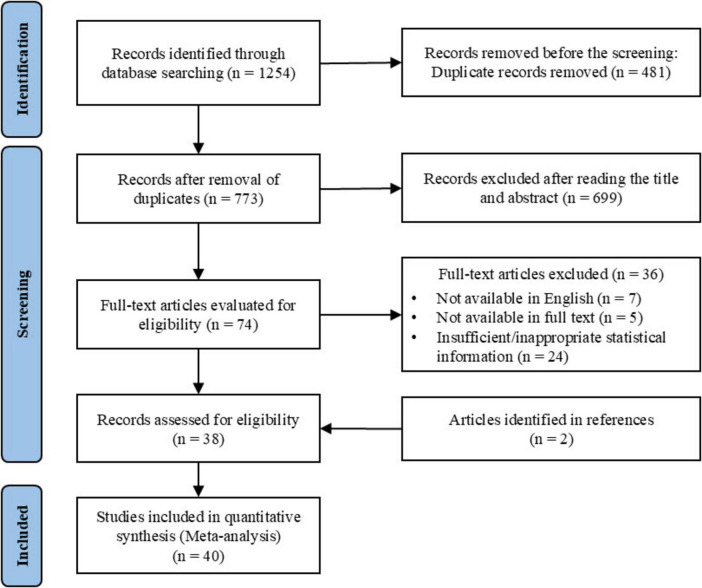
PRISMA flow diagram.

This systematic review and meta-analysis were conducted in accordance with the PRISMA guidelines (Page et al., 2021) and were prospectively registered in the PROSPERO database (registration number: CRD420251054013). A comprehensive literature search was performed across four electronic databases (PubMed, Scopus, Web of Science, and SPORTDiscus) for studies published up to May 20, 2025. Predefined combinations of keywords and Medical Subject Headings (MeSH) were applied, including: (“social support” OR “social identity” OR “social network” OR “family support” OR “friend support” OR “peer support” OR “coach support”) AND (“mental health” OR “wellbeing” OR “anxiety” OR “depress” OR “stress”) AND (“athlete” OR “player”). Detailed search strategies for each database are presented in Supplementary File 1. After the removal of duplicates, titles and abstracts were screened for relevance, followed by full-text assessment based on predefined eligibility criteria. Figure 1 illustrates the study selection process, detailing the stages of screening and inclusion based on the predefined eligibility criteria. Two reviewers independently conducted the screening process, with any disagreements resolved by consultation with a third reviewer.

### Eligibility criteria

Studies were included if they met the following criteria: (a) published in peer-reviewed journals; (b) involved healthy athletes as participants; (c) examined the association between social support and mental health outcomes, including wellbeing, anxiety, depression, or stress; and (d) employed validated instruments to assess both social support and mental health variables. Studies were excluded if they met any of the following conditions: (a) focused on non-athletes or clinical populations (e.g., patients with mental health disorders); (b) did not report on social support or mental health outcomes; (c) did not provide sufficient data to calculate effect sizes.

### Methodological quality assessment and risk of bias

The methodological quality of the included studies was appraised using the Newcastle–Ottawa Scale (NOS) adapted for cross-sectional designs, which assesses study quality across three domains: selection of participants, comparability of study groups, and ascertainment of the outcome. The maximum attainable score is 9. To ensure methodological rigor, only studies scoring greater than 5 points were included in the meta-analysis. Quality assessment was conducted independently by two reviewers, and any discrepancies were resolved through discussion or consultation with a third reviewer. Risk of publication bias was examined through visual inspection of funnel plots and statistically assessed using Egger’s regression test.

### Data Extraction

Mental health outcomes reported across the included studies were categorized into four domains: wellbeing (e.g., assessed using the Psychological Well-Being Scale), anxiety (e.g., Sport Anxiety Scale), depression (e.g., Center for Epidemiologic Studies Depression Scale), and stress (e.g., Perceived Stress Scale). The primary data extracted included measures of association between social support and each mental health outcome, specifically Pearson’s correlation coefficients (r) or standardized regression coefficients (β). In addition, relevant demographic and study-level characteristics were recorded, including participants’ age, gender (female %), athlete level, geographic region, and the instruments used to assess social support and mental health variables. A summary of the extracted data is presented in Table 1.

**TABLE 1:** Characteristics of the included studies.

References	Region	Female%/N	Mean age	Population	Social support measure	Mental health measure	Measure of association	Quality score
Abrahamsen et al., 2008	Norway	48%/143	NG	Elite handball players	Brief-COPE	Trait anxiety: SAS	*r* = −0.21	7
Ackeret et al., 2024	Switzerland	0%/394	18.5	Olympic cardholder athletes	ARSQ	Well-being: MHC Depression: PHQ-9 Stress: PSS-10 Anxiety: GAD	*r* = 0.41 *r* = −0.26 *r* = −0.30 *r* = −0.21	8
Arnold et al., 2018	UK	49%/122	20.5	Talented student athletes	Team: PASS-Q	Stress: OSI-SP	*r* = −0.16	7
Cnen et al., 2021	China	51%/322	NG	University student athletes	Team: ARSQ	Well-being: PWB	*r* = 0.21	7
Cho et al., 2019	USA	22%/368	21.2	University student athletes	Team: SCQ	Anxiety: SAS-2	*r* = −0.19	6
Cho et al., 2020	USA	41%/313	23	University student athletes	Team: PASS-Q	Well-being: flourishing scale	β = 0.294	7
Coussens et al., 2025	UK	46%/534	21.8	University student athletes	Team: PASS-Q	Well-being: WEMWBS	*r* = 0.32	7
Crutcher et al., 2018	USA	69%/204	21.1	University student athletes	SSQ6	Depression: CESD Stress: PSS	*r* = −0.18 *r* = −0.25	6
Cutler and Dwyer, 2020	USA	77%/158	NG	Elite Division I student athletes	Coach: Self-developed scale	Stress: Self-developed scale	*r* = −0.21	5
DeFreese and Smith, 2014	USA	59%/429	19.7	University student athletes	SSQ	Stress: PSS Well-being: SWLS	*r* = −0.39 *r* = 0.45	7
Delfin et al., 2024	USA	0%/93	15.7	High school athletes	Family and friends: PROMIS	Anxiety: PROMIS Depression: PROMIS Stress: PROMIS	*r* = −0.19 *r* = −0.324 *r* = −0.434	6
Forsdyke et al., 2022	UK	45%/150	25.3	Soccer players	PASS-Q	Anxiety: RIAI	*r* = −0.24	7
Glandorf et al., 2022	UK	53%/176	22.9	Female athletes	SSQ	Stress: PSS	β = −0.31	7
Graupensperger et al., 2020	USA	63%/234	19.8	University student athletes	Team: ISSB	Well-being: MHC Depression: PROMIS	*r* = 0.27 *r* = −0.01	6
Hagiwara et al., 2017	USA	49%/204	20.2	Student athletes	Team: SSQ	Depression: SRSA	*r* = −0.06	7
Hagiwara et al., 2021	Japan	0%/402	19.7	University student athletes	Team: SSQ	Depression: SRSA	*r* = −0.20	8
Jeon et al., 2016	South Korea	69%/144	18.7	High school and university athletes	SSQ	Well-being: SWBS	β = 0.28	7
Katagami and Tsuchiya, 2016	Japan	47%/239	19.7	University student athletes	Team: ARSQ	Well-being: PWB	*r* = 0.31	6
Kiliç and Bayköse, 2018	Turkey	51%/236	20.3	Athletes	Family and friends: MPSSS	Anxiety: STAS	*r* = −0.26	7
Kuok et al., 2021	China	56%/84	22.4	Macao elite athletes	ARSQ	Well-being: WEMWBS	*r* = 0.47	7
Latif et al., 2024	Malaysia	41%/298	16.7	SUKMA athletes	MSPSS	Well-being: PWB	*r* = 0.17	6
Lavallée and Flint, 1996	Canada	0%/55	22	University football and rugby players	SSQ	Anxiety: POMS	*r* = −0.32	6
Liu et al., 2023	China	38%/672	20.4	College football athletes	SSQ	Mental health: KPDS	*r* = 0.39	8
Lu et al., 2016	China	27%/218	20	University student athletes	Coach: ARSQ	Stress: CSALSS	r = −0.31	7
Malinauskas and Malinauskiene, 2018	Lithuania	0%/398	23.5	University student athletes	MSPSS	Stress: PSS Well-being: PWB	*r* = −0.30*r* = 0.23	8
Mikesell et al., 2023	USA	100%/3924	20	University student athletes	Family and friends: MSPSS	Stress: PSS Depression: PHQ-2	*r* = −0.25 *r* = −0.30	8
Pan et al., 2022	China	100%/332	21.3	University student athletes	SSQ	Well-being: PWB	Family: *r* = 0.40 Team: *r* = 0.36	7
Peng et al., 2020	China	36%/177	19.1	Provincial athletes	Team: MSPSS	Well-being: SWBS Anxiety: CAAS	*r* = 0.42 *r* = −0.33	7
Poucher et al., 2021	Canada	61%/186	26	Olympics athletes	ARSQ	Stress: PSS Depression: CESD Anxiety: GAD-7 Well-being: SWBS	*r* = −0.21 *r* = −0.20 *r* = −0.13 *r* = 0.17	5
Price and Weiss, 2000	USA	100%/193	16.1	University soccer players	Coach: SSQ	Anxiety: SAS	*r* = 0.02	6
Van Raalte and Posteher, 2019	USA	58%/459	20.2	University student athletes	SSQ	Stress: SLSI	*r* = −0.06	6
Ryska and Yin, 1999	USA	53%/270	16.2	High school team athletes	Coach: SPS	Anxiety: CTA	*r* = −0.22	7
Şenel et al., 2025	Turkey	45%/323	20.1	National judo athletes	Coach: PASS-Q	Well-being: WEMWBS	β = 0.33	7
Simons and Bird, 2023	UK	75%/153	19.5	National student athletes	PASS-Q	Well-being: Sport MHC	*r* = 0.36	6
Solmaz, 2025	Turkey	10%/422	19.8	Professional athletes	MSPSS	Well-being: SWB	*r* = 0.32	8
Sullivan et al., 2020	USA	47%/238	19.7	University student athletes	SSQ	Depression: CESD	*r* = −0.35	6
Sun et al., 2025	China	50%/150	20.6	Professional tennis players	SSQ	Depression: CESD	*r* = −0.41	7
Wezyk, 2011	Poland	39%/75	17.5	Football and volleyball players	SSQ	Anxiety: RCQ	Family: *r* = −0.41 Team: *r* = −0.43 Total: *r* = −0.47	5
Zentgraf et al., 2024	Germany	50%/296	19.2	Squad athletes	PASS-Q	Well-being: PHQ-4	*r* = 0.28	6
Zhao et al., 2022	China	38%/674	NG	College football players	SSQ	Well-being: KPDS	*r* = 0.239	8

ARSQ, athletes’ received support questionnaire; CAAS, coach–athlete attachment scale; CESD, center for epidemiologic study depression scale; CSALSS, college student-athlete life stress scale; CTA, competitive trait anxiety; GAD, general anxiety disorder questionnaire; ISSB, inventory of socially supportive behaviors; KPDS, kessler psychological distress scale; MHC, mental health continuum; MSPSS, multidimensional scale of perceived social support; PASS-Q, perceived available support in sport questionnaire; PHQ, patient health questionnaire; POMS, profile of mood states; PROMIS, patient-reported outcomes measurement information system; PSS, perceived stress scale; PWB, psychological well-being scale; RCQ, reactions to competition questionnaire; RIAI, re-injury anxiety inventory; SAS, sport anxiety scale; SCQ, sport climate questionnaire; SLSI, student-life stress inventory; SPS, social provisions scale; SRSA, stress response scale for athletes; SSP, stressful situations in sport; SSQ, social support questionnaire; SSRS, social support rating scale; STAS, state-trait anxiety scale; SWBS, subjective well-being scale; SWLS, satisfaction with life scale; WEMWBS, warwick-edinburgh mental wellbeing scale.

### Statistical analyses

All meta-analyses were conducted using the *metagen* function from the meta package in R (version 4.3.0). Correlation coefficients (r) were used as the primary effect size. For studies reporting standardized regression coefficients (β), values were converted to r using the following formula:


r=0.98⁢β+0.05⁢λ

Where λ was set to 1 if β was positive and 0 if negative (Peterson and Brown, 2005). To stabilize variance and improve the precision of pooled estimates, Fisher’s z-transformation was applied to all *r* values prior to analysis (Lei et al., 2018), with back-transformation performed to facilitate interpretation. A random-effects model was employed to account for heterogeneity across studies. *I*^2^ statistics were used to assess heterogeneity, with values ≥50% indicating substantial heterogeneity across studies. Effect sizes were interpreted as small (*r* < 0.30), moderate (0.30 ≤ *r* < 0.50), or large (*r* ≥ 0.50), with statistical significance defined as *p* < 0.05.

Subgroup meta-analyses were conducted to examine the associations between social support and mental health outcomes across three categories: (1) overall social support, (2) support from sport team members (e.g., teammates and coaches), and (3) support from family and friends. Additionally, moderation analyses were conducted to assess the moderating role of gender and athlete level in the strength and direction of the relationships between social support and mental health. Gender composition was defined by the proportion of female participants in each study, grouped into three categories: ≤40%, 40%–60%, and ≥60%. Athlete level was divided into professional (e.g., Olympic, national, and provincial level athletes) and amateur groups (e.g., student athletes and participants in campus or amateur competitions).

## Results

### Study selection and characteristics

A total of 1,254 studies were initially identified through keyword searches. After eliminating duplicates, 773 articles remained for further screening. Title and abstract review resulted in 74 studies, which were then evaluated for full-text eligibility. Based on the eligibility criteria, 38 studies were selected for inclusion. Additionally, two more studies were identified through reference list reviews, bringing the total number of studies included in the meta-analysis to 40. The full screening process is shown in Figure 1.

Table 1 summarizes the key characteristics of the studies included in the meta-analysis. The majority of the studies included a mixed-gender sample, with participants aged 16–26 years, and most studies reporting an average age between 18 and 23 years. Participants were categorized into professional athletes (e.g., Olympic, elite, national-level) and non-professional athletes (e.g., university and high school athletes). The most commonly used social support measures were the Social Support Questionnaire and the Perceived Athlete Social Support Questionnaire. Mental health outcomes were assessed using a variety of scales, with common measures for anxiety (e.g., Sport Anxiety Scale, Generalized Anxiety Disorder), depression (e.g., Center for Epidemiologic Study Depression Scale, Patient-Reported Outcomes Measurement Information System), stress (e.g., Perceived Stress Scale), and well-being (e.g., Warwick-Edinburgh Mental Well-being Scale).

### Methodological quality assessment and risk of bias

The methodological quality of the included studies was assessed using the NOS for cross-sectional studies. Of the 40 studies, 17 were rated as moderate quality (5–6 points), while 23 were rated as high quality (7–8 points), indicating an overall moderate to high methodological quality. A detailed NOS evaluation is provided in Supplementary File 2. The funnel plot indicated a generally symmetrical distribution, suggesting no significant bias (Supplementary File 3). Egger’s test for publication bias revealed no significant bias in the meta-analysis examining the relationship between overall social support and well-being (*t* = −0.21, *b* = −0.49, *p* = 0.83). Due to the small number of studies (fewer than 10) included in the other meta-analyses, Egger’s test was not applicable for those analyses.

### Meta-analysis results

13 studies, involving a total of 4,482 participants, examined the relationship between total social support and well-being, revealing a significant moderate effect (*r* = 0.31, 95% CI [0.26, 0.36], *p* < 0.001). Seven studies, with 1,273 participants, assessed the relationship between overall social support and anxiety, demonstrating a significant small negative effect (*r* = −0.22, 95% CI [−0.27, −0.17], *p* < 0.001). Eight studies, comprising 2,404 participants, explored the relationship between overall social support and stress, showing a significant small negative effect (*r* = −0.25, 95% CI [−0.32, −0.18], *p* < 0.001). Finally, five studies involving 1,172 participants examined the link between overall social support and depression, revealing a significant small negative effect (*r* = −0.27, 95% CI [−0.35, −0.19], *p* < 0.001). Detailed forest plots visualizing the meta-analytic findings can be found in Supplementary File 4.

Subgroup analysis revealed no significant differences in the relationships between team support and well-being, and between family-friends support and well-being. Similarly, no significant differences were found in the relationships between team support, family-friends support, and anxiety or stress. However, a significant difference was observed in the relationship between social support and depression (*p* < 0.01), with family-friends support showing a stronger negative association with depression. The detailed meta-analysis and subgroup analysis results are provided in Table 2. Furthermore, moderation analyses revealed no significant moderating effects of gender composition or athlete level on the relationships between overall social support and various mental health outcomes, including wellbeing, anxiety, and stress (see Supplementary File 5).

**TABLE 2:** Correlations between social support and mental health indicators.

Mental health indicators	Social support type	*k*	*r* [95% CI]	p1	I^2^ (%)	p2
Well-being	Total social support	13	0.31 [0.26, 0.36]	***	74.2	0.79
	Team support	7	0.29 [0.24, 0.33]	***	40.8	
	Family-friends support	2	0.27 [0.06, 0.44]	*	86.4	
Anxiety	Total social support	7	−0.22 [−0.27, −0.17]	***	27.4	0.34
	Team support	5	−0.22 [−0.35, −0.08]	**	77.5	
	Family-friends support	3	−0.30 [−0.40, −0.17]	***	0	
Stress	Total social support	8	−0.25 [−0.32, −0.18]	***	75.7	0.74
	Team support	3	−0.24 [−0.32, −0.15]	***	2.8	
	Family-friends support	4	−0.21 [−0.31, −0.09]	***	81.6	
Depression	Total social support	5	−0.27 [−0.35, −0.19]	***	55.7	**
	Team support	3	−0.10 [−0.21, 0.02]	0.10	65.2	
	Family-friends support	2	−0.29 [−0.32, −0.26]	***	0	

p1 indicates the significance level of the meta-analysis results. p2 represents the significance level of the comparison between team support and family-friends support.

*** denotes *p* < 0.001,

** denotes *p* < 0.01.

## Discussion

This meta-analysis shows a significant positive relationship between social support and well-being in athletes, along with a negative relationship between social support and anxiety, stress, and depression. Family-friends support was found to have a stronger negative relationship with depression compared to team support. Furthermore, moderation analyses revealed that gender and athlete level did not significantly moderate these relationships. These findings underscore the important role of social support in the mental health of athletes, particularly in reducing anxiety, stress, and depression, with consistent findings across different genders and athlete levels.

The findings of this meta-analysis align with previous research in both general and clinical populations, which highlight social support as a crucial buffering mechanism for mitigating psychological stress (Bedaso et al., 2021; Wang et al., 2018; Zhou and Cheng, 2022). It has been well-established in the literature that social support enhances resilience, alleviates the negative impacts of stress, and improves emotional regulation (Acoba, 2024). However, the context for athletes is distinctly different. Athletes not only face the typical psychological challenges of daily life but also deal with sport-specific stressors such as performance pressure, competition anxiety, and the psychological consequences of injuries (Stevens et al., 2024). These factors make athletes’ mental health particularly vulnerable, especially in high-competition environments, where the need for psychological support becomes even more critical (Küttel and Larsen, 2020).

Our study indicates a stronger association between support from family and friends and the reduction of depressive symptoms compared to support from coaches or teammates. This finding underscores the unique role of intimate and stable relationships in addressing mental health challenges. The support from family and friends often extends beyond the athletic domain and provides stable emotional regulation resources that are essential for managing persistent negative emotions (Lisinskiene and Lochbaum, 2022; Mira et al., 2023). In contrast, team support tends to focus on performance and goal achievement (González-García et al., 2022), and while it positively impacts overall well-being, anxiety, and stress, its direct emotional support for mental health is likely weaker. These results suggest that targeted psychological health interventions should not only focus on strengthening team dynamics but should also prioritize personal and familial support networks to address emotional and psychological needs more effectively.

Our meta-analysis found no significant moderating effects of gender or athlete level on the relationship between social support and mental health outcomes. This suggests that, within the scope of this study, the association between social support and mental health outcomes (e.g., wellbeing, anxiety, and stress) remains consistent across both genders and athlete levels. However, the lack of significant moderation may be due to the limited sample sizes and study heterogeneity, which could have reduced statistical power. Future research with larger, more homogeneous samples could provide clearer insights into the potential moderating roles of these factors.

Several limitations of this meta-analysis warrant cautious interpretation of the findings. First, substantial heterogeneity was observed across studies, which may be partly attributed to differences in sample characteristics, sport types, and the instruments used to assess social support and mental health outcomes. Additionally, the limited number of studies available for certain psychological outcomes (e.g., anxiety and depression) may have reduced the stability and generalizability of the pooled estimates. Finally, the heterogeneity of assessment tools across studies compromised the comparability of findings and hindered the examination of instrument-specific effects. Future research should utilize standardized and validated instruments to improve measurement consistency, and report disaggregated data to facilitate subgroup analyses.

## Conclusion

This meta-analysis demonstrates that social support is crucial in enhancing athletes’ mental health by improving well-being and reducing symptoms of anxiety, depression, and stress. Notably, support from family and friends has a stronger association with alleviating depressive symptoms compared to team-based support, emphasizing the value of intimate, stable relationships. These findings emphasize the importance of incorporating multiple sources of social support in mental health strategies for athletes to effectively address their psychological needs.

## Data Availability

The original contributions presented in this study are included in this article/Supplementary material, further inquiries can be directed to the corresponding authors.

## References

[B1] Abrahamsen F. Roberts G. Pensgaard A. Ronglan L. (2008). Perceived ability and social support as mediators of achievement motivation and performance anxiety. *Scand. J. Med. Sci. Sports* 18 810–821. 10.1111/j.1600-0838.2007.00707.x 18208425

[B2] Ackeret N. Röthlin P. Horvath S. (2024). Factors contributing to elite athletes’ mental health in the junior-to-senior transition: A mixed methods study. *Psychol. Sport Exerc.* 73:102645. 10.1016/j.psychsport.2024.102645 38608852

[B3] Acoba E. F. (2024). Social support and mental health: the mediating role of perceived stress. *Front. Psychol.* 15:1330720. 10.3389/fpsyg.2024.1330720 38449744 PMC10915202

[B4] Arnold R. Edwards T. Rees T. (2018). Organizational stressors, social support, and implications for subjective performance in high-level sport. *Psychol. Sport Exerc.* 39 204–212. 10.1016/j.psychsport.2018.08.010

[B5] Bedaso A. Adams J. Peng W. Sibbritt D. (2021). The relationship between social support and mental health problems during pregnancy: A systematic review and meta-analysis. *Reproductive Health* 18 1–23. 10.1186/s12978-021-01209-5 34321040 PMC8320195

[B6] Cho H. Yi Tan H. Lee E. (2020). Importance of perceived teammate support as a predictor of student-athletes’ positive emotions and subjective well-being. *Int. J. Sports Sci. Coaching* 15 364–374. 10.1177/1747954120919720

[B7] Cho S. Choi H. Kim Y. (2019). The relationship between perceived coaching behaviors, competitive trait anxiety, and athlete burnout: A cross-sectional study. *Int. J Environ. Res. Public Health* 16:1424. 10.3390/ijerph16081424 31010073 PMC6517960

[B8] Cnen T.-W. Chiu Y.-C. Hsu Y. (2021). Perception of social support provided by coaches, optimism/pessimism, and psychological well-being: Gender differences and mediating effect models. *Int. J. Sports Sci. Coach.* 16 272–280. 10.1177/1747954120968649

[B9] Cohen S. Wills T. A. (1985). Stress, social support, and the buffering hypothesis. *Psychol. Bull.* 98:310. 10.1037/0033-2909.98.2.310 3901065

[B10] Coussens A. H. Stone M. J. Donachie T. C. (2025). Coach–athlete relationships, self-confidence, and psychological wellbeing: The role of perceived and received coach support. *Eur. J. Sport Sci.* 25:e12226. 10.1002/ejsc.12226 39587818 PMC11680554

[B11] Crutcher B. Moran R. N. Covassin T. (2018). Examining the relationship between social support satisfaction and perceived stress and depression in athletic training students. *Athletic Training Educ. J.* 13 168–174. 10.4085/1302168

[B12] Cutler B. A. Dwyer B. (2020). Student-athlete perceptions of stress, support, and seeking mental health services. *J. Issues Intercollegiate Athletics* 13:16. 10.1016/s0891-5245(05)80031-8 7629682

[B13] Daley M. M. Shoop J. Christino M. A. (2023). Mental health in the specialized athlete. *Curr Rev Musculoskeletal Med.* 16 410–418. 10.1007/s12178-023-09851-1 37326758 PMC10427563

[B14] DeFreese J. D. Smith A. L. (2014). Athlete social support, negative social interactions, and psychological health across a competitive sport season. *J. Sport Exerc. Psychol.* 36 619–630. 10.1123/jsep.2014-0040 25602144

[B15] Delfin D. Wallace J. Baez S. Karr J. E. Terry D. P. Hibbler T. (2024). Social support, stress, and mental health: examining the stress-buffering hypothesis in adolescent football athletes. *J. Athletic Training* 59 499–505. 10.4085/1062-6050-0324.23 38014810 PMC11127675

[B16] Forsdyke D. Madigan D. Gledhill A. Smith A. (2022). Perceived social support, reinjury anxiety, and psychological readiness to return to sport in soccer players. *J. Sport Rehabil.* 31 749–755. 10.1123/jsr.2021-0181 35405636

[B17] Glandorf H. L. Coffee P. Madigan D. J. (2022). Team identification and athlete burnout: Testing longitudinal serial mediation via perceived support and stress. *Psychol. Sport Exerc.* 63:102292. 10.1016/j.psychsport.2022.102292

[B18] González-García H. Martinent G. Nicolas M. (2022). Relationships between coach’s leadership, group cohesion, affective states, sport satisfaction and goal attainment in competitive settings. *Int. J. Sports Sci. Coach.* 17, 244–253. 10.1177/17479541211053229

[B19] Graupensperger S. Benson A. J. Kilmer J. R. Evans M. B. (2020). Social (un) distancing: Teammate interactions, athletic identity, and mental health of student-athletes during the COVID-19 pandemic. *J. Adolesc. Health* 67 662–670. 10.1016/j.jadohealth.2020.08.001 32943294 PMC7489994

[B20] Hagiwara G. Iwatsuki T. Isogai H. van Raalte J. L. Brewer B. W. (2017). Relationships among sports helplessness, depression, and social support in American college student-athletes. *J. Phys. Educ. Sport* 17:753. 10.7752/jpes.2017.02114 33809896

[B21] Hagiwara G. Tsunokawa T. Iwatsuki T. Shimozono H. Kawazura T. (2021). Relationships among student-athletes’ identity, mental health, and social support in Japanese student-athletes during the COVID-19 pandemic. *Int. J. Environ. Res. Public Health* 18:7032. 10.3390/ijerph18137032 34209463 PMC8297159

[B22] Hartley C. Hartley C. Coffee P. (2023). “The influence of social support on athletes,” in: *Social Psychology in Sport*, eds Davis L. Keegan R. Jowett S.. (Champaign, IL: Human Kinetics).

[B23] Haugen E. (2022). Athlete mental health & psychological impact of sport injury. *Operative Techniques Sports Med.* 30:150898. 10.1016/j.otsm.2022.150898

[B24] Jeon H. Lee K. Kwon S. (2016). Investigation of the structural relationships between social support, self-compassion, and subjective well-being in Korean elite student athletes. *Psychol. Rep.* 119 39–54. 10.1177/0033294116658226 27381414

[B25] Katagami E. Tsuchiya H. (2016). Effects of social support on athletes’ psychological well-being: The correlations among received support, perceived support, and personality. *Psychology* 7:1741. 10.4236/psych.2016.713163

[B26] Keyes C. L. (2002). The mental health continuum: From languishing to flourishing in life. *J. Health Soc. Behav.* 43 207–222. 10.2307/3090197 12096700

[B27] Kiliç T. Bayköse N. (2018). The mediator role of family support in relation between continuous anxiety and mental toughness in athletes. *Turkish Online J. Educ. Technol.* 17, 769–773.

[B28] Kuok A. C. Chio D. K. Pun A. C. (2021). Elite athletes’ mental well-being and life satisfaction: a study of elite athletes’ resilience and social support from an Asian unrecognised National Olympic Committee. *Health Psychol. Rep.* 10 302–312. 10.5114/hpr.2021.107073 38084133 PMC10670795

[B29] Küttel A. Larsen C. H. (2020). Risk and protective factors for mental health in elite athletes: A scoping review. *Int. Rev. Sport Exerc. Psychol.* 13 231–265. 10.1080/1750984X.2019.1689574

[B30] Latif R. A. Majeed H. A. Tumijan W. Tajri A. A. Rajli M. A. Hidayat Y. (2024). Enhancing athletic well-being: Unravelling the impact of social support. *Information Manag. Bus. Rev.* 16 248–256. 10.22610/imbr.v16i3(I).3796

[B31] Lavallée L. Flint F. (1996). The relationship of stress, competitive anxiety, mood state, and social support to athletic injury. *J. Athletic Training* 31 296–299. PMC1318911 16558413

[B32] Lei H. Cui Y. Zhou W. (2018). Relationships between student engagement and academic achievement: A meta-analysis. *Soc. Behav. Pers. Int. J.* 46 517–528. 10.2224/sbp.7054

[B33] Lisinskiene A. Lochbaum M. (2022). The coach–athlete–parent relationship: The importance of the sex, sport type, and family composition. *Int. J. Environ. Res. Public Health* 19:4821. 10.3390/ijerph19084821 35457690 PMC9025112

[B34] Liu Z. Zhao X. Zhao L. Zhang L. (2023). Relationship between perceived social support and mental health among Chinese college football athletes: A moderated mediation model. *BMC Psychol.* 11:329. 10.1186/s40359-023-01357-2 37822005 PMC10568796

[B35] Lu F. J. Lee W. P. Chang Y.-K. Chou C.-C. Hsu Y.-W. Lin J.-H. (2016). Interaction of athletes’ resilience and coaches’ social support on the stress-burnout relationship: A conjunctive moderation perspective. *Psychol. Sport Exerc.* 22 202–209. 10.1016/j.psychsport.2015.08.005

[B36] Mahindru A. Patil P. Agrawal V. (2023). Role of physical activity on mental health and well-being: A review. *Cureus* 15:e33475. 10.7759/cureus.33475 36756008 PMC9902068

[B37] Malinauskas D. R. Malinauskiene V. (2018). The mediation effect of perceived social support and perceived stress on the relationship between emotional intelligence and psychological wellbeing in male athletes. *J. Hum. Kinetics* 65 291–303. 10.2478/hukin-2018-0017 30687440 PMC6341946

[B38] Mikesell M. Petrie T. A. Chu T. L. A. Moore E. W. G. (2023). The relationship of resilience, self-compassion, and social support to psychological distress in women collegiate athletes during COVID-19. *J. Sport Exerc. Psychol.* 45 224–233. 10.1123/jsep.2022-0262 37474120

[B39] Mira T. Jacinto M. Costa A. M. Monteiro D. Diz S. Matos R. (2023). Exploring the relationship between social support, resilience, and subjective well-being in athletes of adapted sport. *Front. Psychol.* 14:1266654. 10.3389/fpsyg.2023.1266654 38144980 PMC10748803

[B40] Nuetzel B. (2023). Coping strategies for handling stress and providing mental health in elite athletes: A systematic review. *Front. Sports Act. Living* 5:1265783. 10.3389/fspor.2023.1265783 38033656 PMC10687549

[B41] Page M. J. Mckenzie J. E. Bossuyt P. M. Boutron I. Hoffmann T. C. Mulrow C. D. (2021). The PRISMA 2020 statement: An updated guideline for reporting systematic reviews. *bmj* 372:n71. 10.1136/bmj.n71 33782057 PMC8005924

[B42] Pan H.-W. Huang W.-Y. Wu C.-E. (2022). Research on the relationships among the gender consciousness, social support, and wellbeing in Taiwan college female athletes. *SAGE Open* 12:21582440221097895. 10.1177/21582440221097895

[B43] Peng J. Zhang J. Zhao L. Fang P. Shao Y. (2020). Coach–athlete attachment and the subjective well-being of athletes: A multiple-mediation model analysis. *Int. J. Environ. Res. Public Health* 17:4675. 10.3390/ijerph17134675 32610591 PMC7369865

[B44] Peterson R. A. Brown S. P. (2005). On the use of beta coefficients in meta-analysis. *J. Appl. Psychol.* 90 175–181. 10.1037/0021-9010.90.1.175 15641898

[B45] Poucher Z. Tamminen K. Sabiston C. Cairney J. Kerr G. (2021). Prevalence of symptoms of common mental disorders among elite Canadian athletes. *Psychol. Sport Exerc.* 57:102018. 10.1016/j.psychsport.2021.102018

[B46] Price M. S. Weiss M. R. (2000). Relationships among coach burnout, coach behaviors, and athletes’ psychological responses. *Sport Psychol.* 14 391–409. 10.1123/tsp.14.4.391

[B47] Reardon C. L. (2021). The mental health of athletes: Recreational to elite. *Curr. Sports Med. Rep*. 20, 631–637. 10.1249/JSR.0000000000000916 34882119

[B48] Ryska T. A. Yin Z. (1999). Testing the buffering hypothesis: Perceptions of coach support and pre-competitive anxiety among male and female high school athletes. *Curr. Psychol.* 18 381–393. 10.1007/s12144-999-1011-5

[B49] Şenel E. Küttel A. Adiloğullari İ Jowett S. (2025). Psychological predictors of mental well-being in Judo athletes: Exploring the impacts of the coach-athlete relationship, social support, and psychological safety. *Psychol. Sport Exerc.* 79:102850. 10.1016/j.psychsport.2025.102850 40185177

[B50] Simons E. E. Bird M. D. (2023). Coach-athlete relationship, social support, and sport-related psychological well-being in National collegiate athletic association division I student-athletes. *J. Study Sports Athletes Educ.* 17 191–210. 10.1080/19357397.2022.2060703

[B51] Solmaz S. (2025). Enhancing subjective well-being in young professional athletes: The role of self-esteem and perceived social support in moderating neuroticism. *SAGE Open* 15:21582440251323673. 10.1177/21582440251323673

[B52] Stevens M. Cruwys T. Olive L. Rice S. (2024). Understanding and improving athlete mental health: A social identity approach. *Sports Med.* 54 837–853. 10.1007/s40279-024-01996-4 38407748 PMC11052891

[B53] Sullivan M. Moore M. Blom L. C. Slater G. (2020). Relationship between social support and depressive symptoms in collegiate student athletes. *J. Study Sports Athletes Educ.* 14 192–209. 10.1080/19357397.2020.1768034

[B54] Sun R. Li T. Li M. Meng L. (2025). The effects of tennis on depressive symptoms and pro-social behaviors in university students: The mediating role of appreciative social support. *Front. Psychol.* 16:1428977. 10.3389/fpsyg.2025.1428977 40181900 PMC11965648

[B55] Van Raalte L. J. Posteher K. A. (2019). Examining social support, self-efficacy, stress, and performance, in US Division I collegiate student-athletes’ academic and athletic lives. *J. Study Sports Athletes Educ.* 13 75–96. 10.1080/19357397.2019.1635419

[B56] Wang J. Mann F. Lloyd-Evans B. Ma R. Johnson S. (2018). Associations between loneliness and perceived social support and outcomes of mental health problems: A systematic review. *BMC Psychiatry* 18:156. 10.1186/s12888-018-1736-5 29843662 PMC5975705

[B57] Wezyk A. (2011). Relationships between competitive anxiety, social support and self-handicapping in youth Sport. *Biomed. Hum. Kinetics* 3 72–77. 10.2478/v10101-011-0016-3

[B58] Zentgraf K. Musculus L. Reichert L. Will L. Roffler A. Hacker S. (2024). Advocating individual-based profiles of elite athletes to capture the multifactorial nature of elite sports performance. *Sci. Rep.* 14:26351. 10.1038/s41598-024-76977-8 39487215 PMC11530532

[B59] Zhao L. Liu Z. Zhang L. (2022). The effect of the perceived social support on mental health of Chinese college soccer players during the COVID-19 lockdown: The chain mediating role of athlete burnout and hopelessness. *Front. Psychol.* 13:1001020. 10.3389/fpsyg.2022.1001020 36438322 PMC9691843

[B60] Zhou Z. Cheng Q. (2022). Relationship between online social support and adolescents’ mental health: A systematic review and meta-analysis. *J. Adolesc.* 94 281–292. 10.1002/jad.12031 35390193

