# Supplementary material for: The relationship between social support and mental health in athletes: a systematic review and meta-analysis
Source: Front Psychol. 2025 Sep 3;16:1642886. doi: 10.3389/fpsyg.2025.1642886 (PMC12442422; doi:10.3389/fpsyg.2025.1642886)
Supplement: Supplementary file 1 [file Data_Sheet_1.pdf]

S1 File. Search Alert

Pubmed (73)
(("social support"[Title/Abstract] OR "social identity"[Title/Abstract] OR "social network"[Title/Abstract] OR "family support"[Title/Abstract] OR "friend support"[Title/Abstract] OR "peer support"[Title/Abstract] OR "coach support"[Title/Abstract]) AND ("mental health"[Title/Abstract] OR "wellbeing"[Title/Abstract] OR "anxiety"[Title/Abstract] OR "depress"[Title/Abstract] OR "stress"[Title/Abstract])) AND ("athlete"[Title/Abstract] OR "player"[Title/Abstract])

SCOPUS (636)
[Article title, Abstract, Keywords] ("social support" OR "social identity" OR "social network" OR "family support" OR "friend support" OR "peer support" OR "coach support"); AND [Article title, Abstract, Keywords] ("mental health" OR "wellbeing" OR "anxiety" OR "depress" OR "stress") AND ("athlete" OR "player")

SPORTDiscus (108)
Search Alert: ("AB ("social support" OR "social identity" OR "social network" OR "family support" OR "friend support" OR "peer support" OR "coach support") AND AB ("mental health" OR "wellbeing" OR "anxiety" OR "depress" OR "stress") AND ("athlete" OR "player"))

Web of Science (437)
[Topic] ("social support" OR "social identity" OR "social network" OR "family support" OR "friend support" OR "peer support" OR "coach support") AND [Topic] ("mental health" OR "wellbeing" OR "anxiety" OR "depress" OR "stress") AND ("athlete" OR "player")

S2 File. Newcastle-Ottawa Scale (Adapted for Cross-Sectional Studies)

Table S1. Newcastle-Ottawa Scale (Adapted for Cross-Sectional Studies)

**Studies**	**Selection**				**Comparability**	**Outcome**		**Total NOS**
	**1**	**2**	**3**	**4**	**5**	**6**	**7**	
Abrahamsen 2008	1	0	1	2	1	1	1	7
Ackeret 2024	1	1	1	2	1	1	1	8
Arnold 2018	1	0	1	2	1	1	1	7
Chen 2021	1	0	1	2	1	1	1	7
Cho 2019	1	0	0	2	1	1	1	6
Cho 2020	1	0	1	2	1	1	1	7
Coussens 2025	1	1	0	2	1	1	1	7
Crutcher 2018	1	0	1	2	0	1	1	6
Cutler 2020	1	0	1	1	0	1	1	5
DeFreese 2014	1	1	0	2	1	1	1	7
Delfin 2024	1	0	1	2	0	1	1	6
Forsdyke 2022	1	0	1	2	1	1	1	7
Glandorf 2022	1	0	1	2	1	1	1	7
Graupensperger 2020	1	0	1	1	1	1	1	6
Hagiwara 2017	1	0	1	2	1	1	1	7
Hagiwara 2021	1	1	1	2	1	1	1	8
Jeon 2016	1	0	1	2	1	1	1	7
Katagami 2016	1	0	1	2	0	1	1	6
Kilic 2018	1	0	1	2	1	1	1	7
Kuok 2022	1	0	1	2	1	1	1	7
Latif 2024	1	0	1	2	0	1	1	6
Lavallee 1996	1	0	1	2	0	1	1	6
Liu 2023	1	1	1	2	1	1	1	8
Lu 2016	1	0	1	2	1	1	1	7
Malinauskas 2018	1	1	1	2	1	1	1	8
Mikesell 2023	1	1	1	2	1	1	1	8
Pan 2022	1	0	1	2	1	1	1	7
Peng 2020	1	0	1	2	1	1	1	7
Poucher 2021	1	0	0	2	0	1	1	5
Price 2000	1	0	1	2	0	1	1	6
Raalte 2019	1	1	0	2	0	1	1	6
Ryska 1999	1	0	1	2	1	1	1	7
Senel 2025	1	0	1	2	1	1	1	7
Simons 2023	1	0	1	2	0	1	1	6
Solmaz 2025	1	1	1	2	1	1	1	8
Sullivan 2020	1	1	0	2	0	1	1	6
Sun 2025	1	0	1	2	1	1	1	7
Wezyk 2011	1	0	0	2	0	1	1	5

Zentgraf 2024	1	1	0	2	0	1	1	6
Zhao 2022	1	1	1	2	1	1	1	8

Note. 1, Representativeness of the sample; 2, Sample size; 3, Non-respondents; 4, Ascertainment of the exposure; 5, Comparability; 6, Assessment of the outcome; 7, Statistical test. Studies with NOS scores <5 have been excluded.

S3 File. Funnel plots

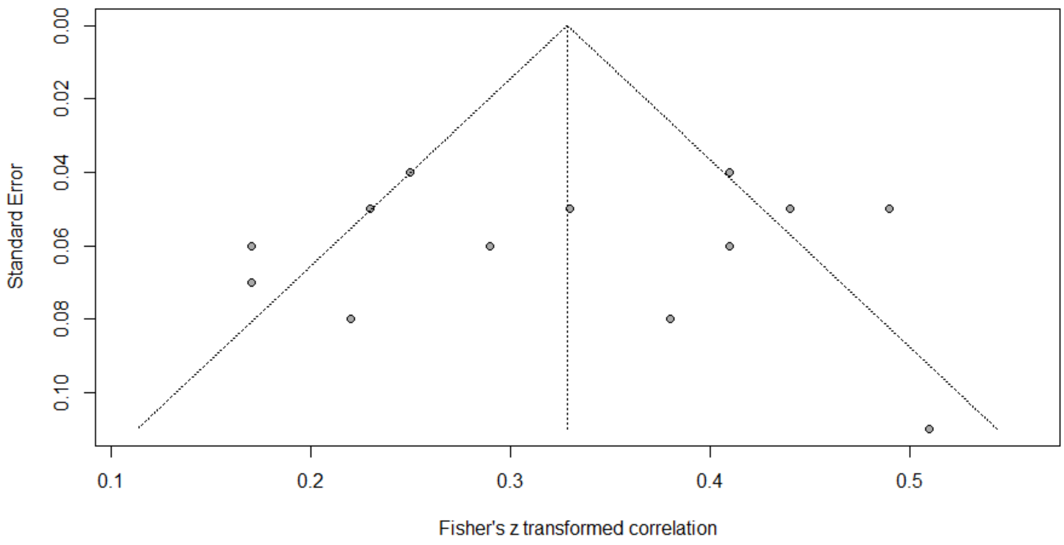

Figure S1. Funnel plot of the association between social support and well-being

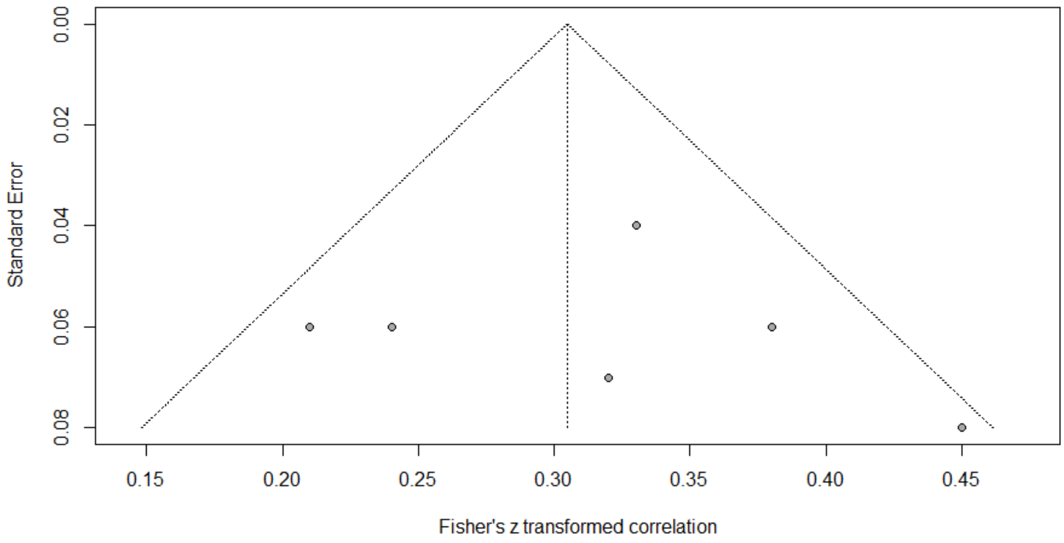

Figure S2. Funnel plot of the association between team support and well-being

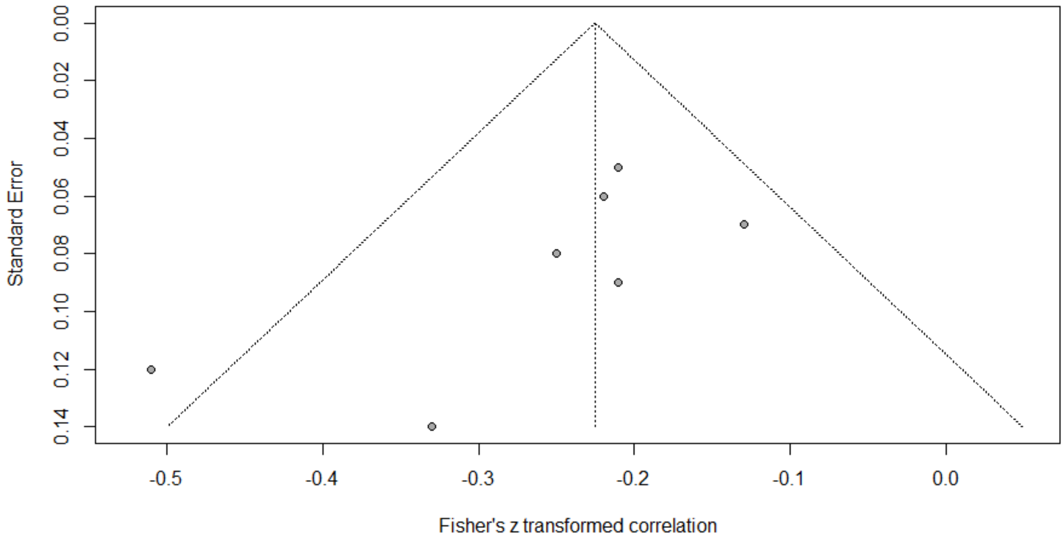

Figure S3. Funnel plot of the association between social support and anxiety

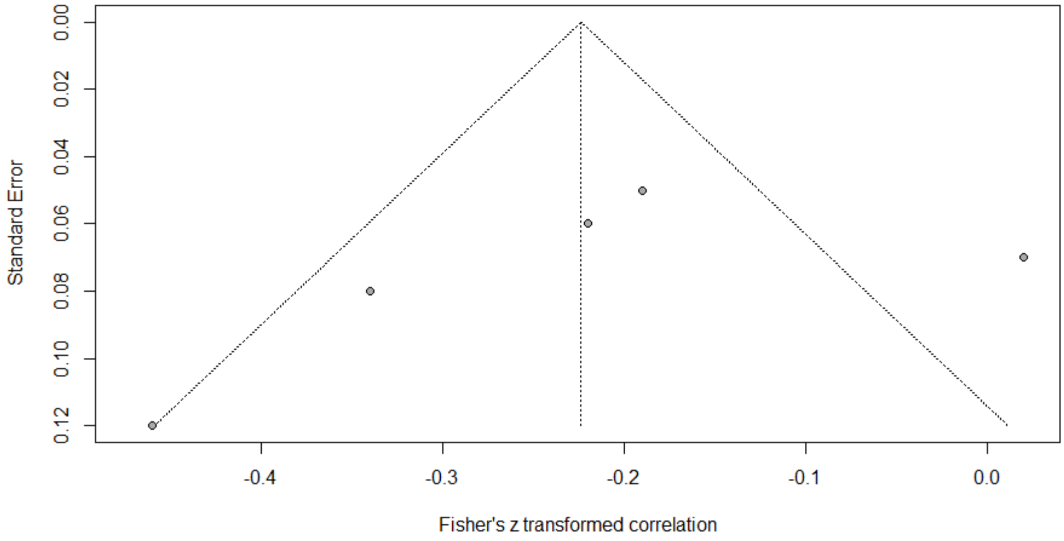

Figure S4. Funnel plot of the association between team support and anxiety

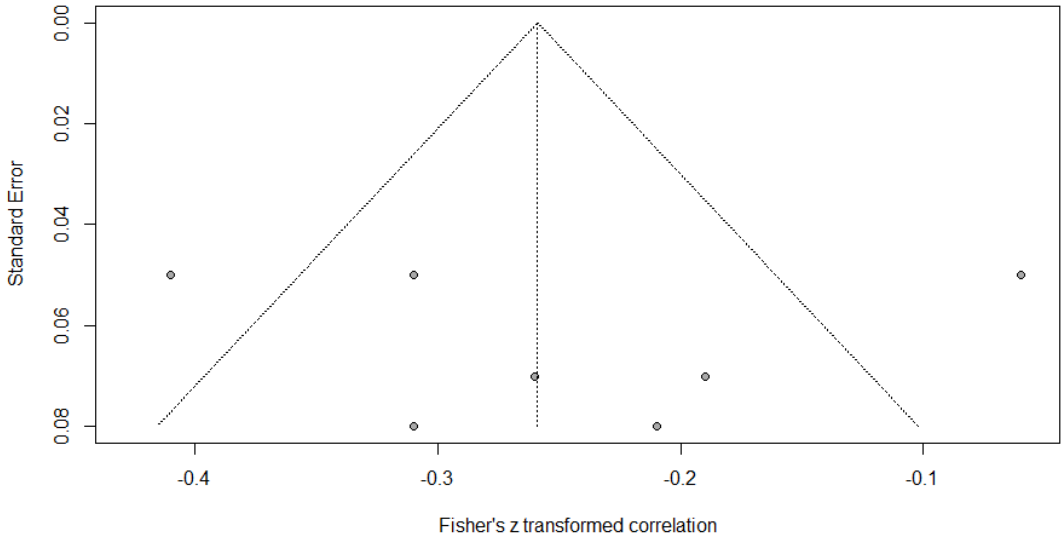

Figure S5. Funnel plot of the association between social support and stress

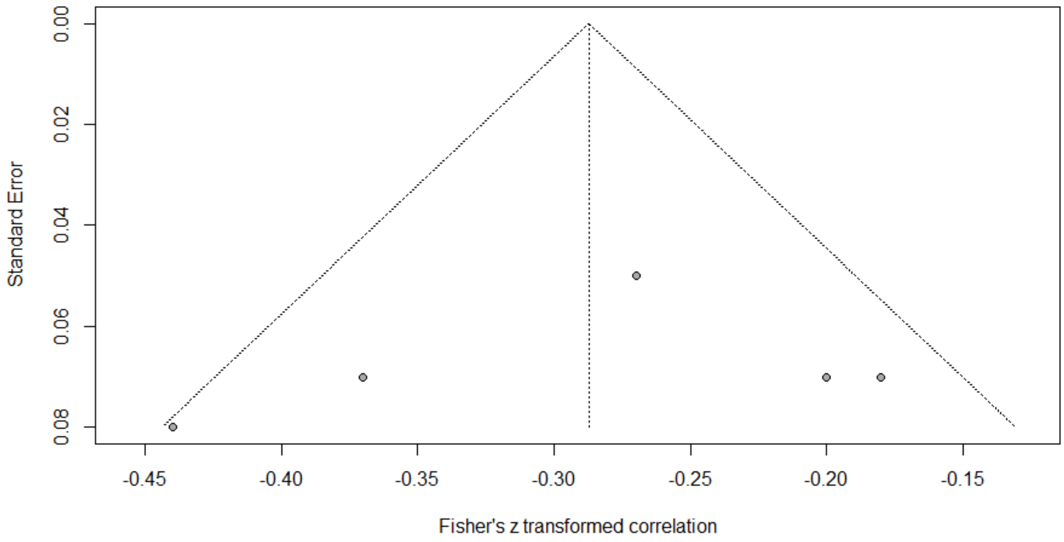

Figure S6. Funnel plot of the association between social support and depression

S4 File. Forest plots

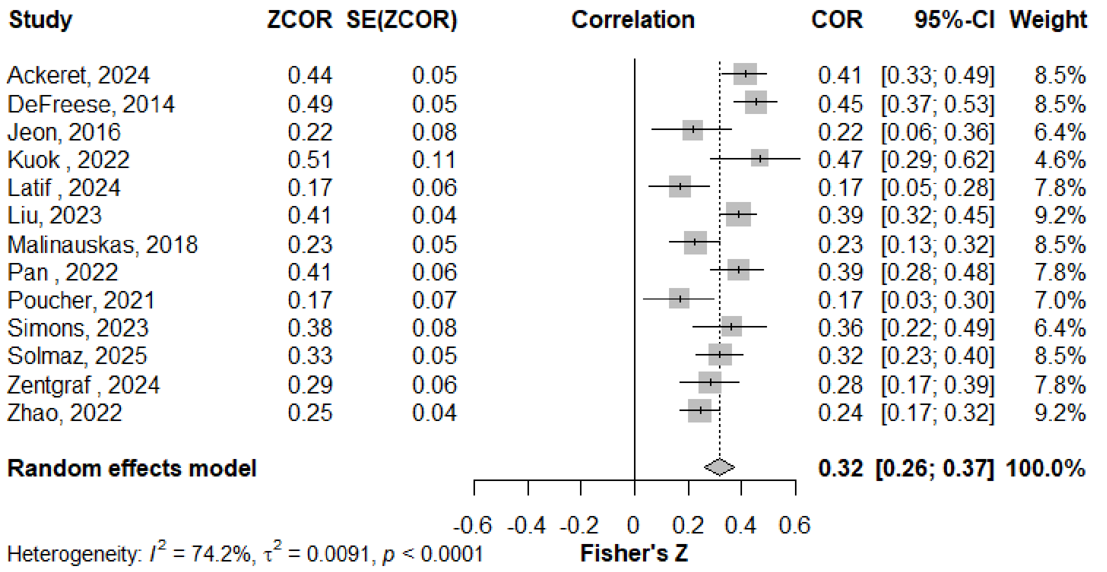

Figure S12. Forest plot of the association between social support and wellbeing

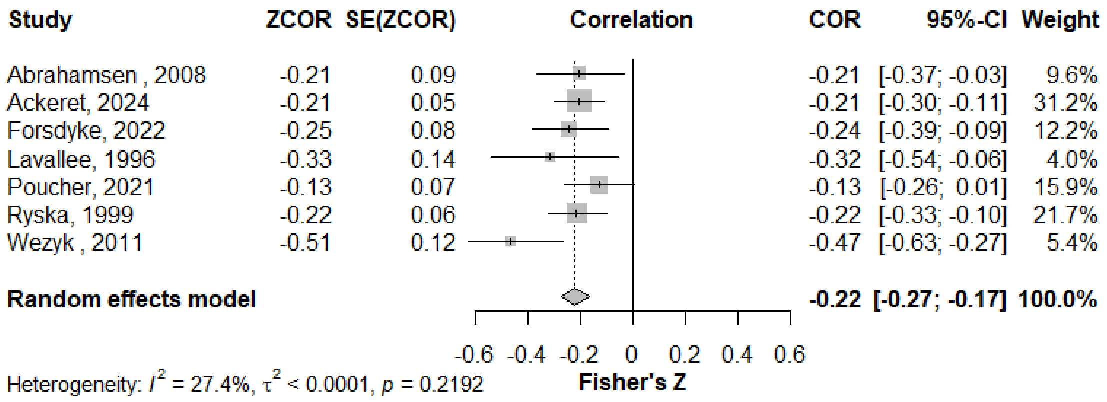

Figure S13. Forest plot of the association between social support and anxiety

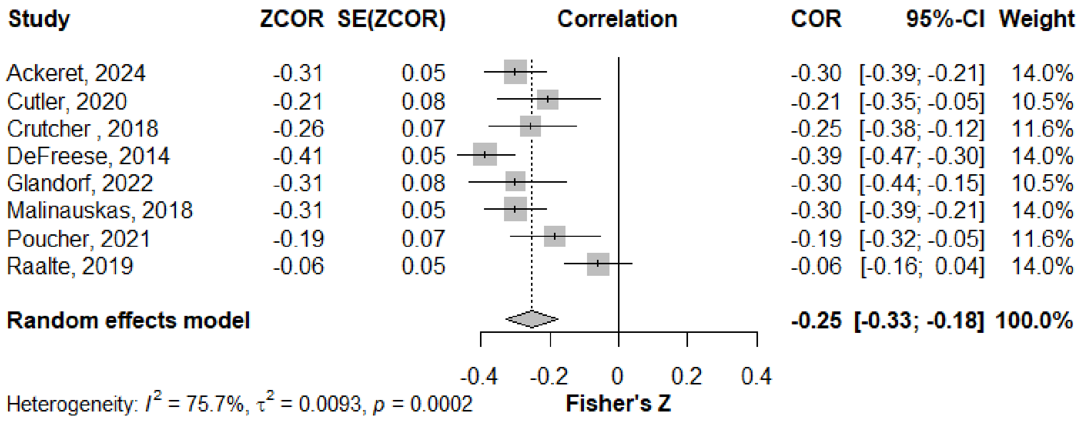

Figure S14. Forest plot of the association between social support and stress

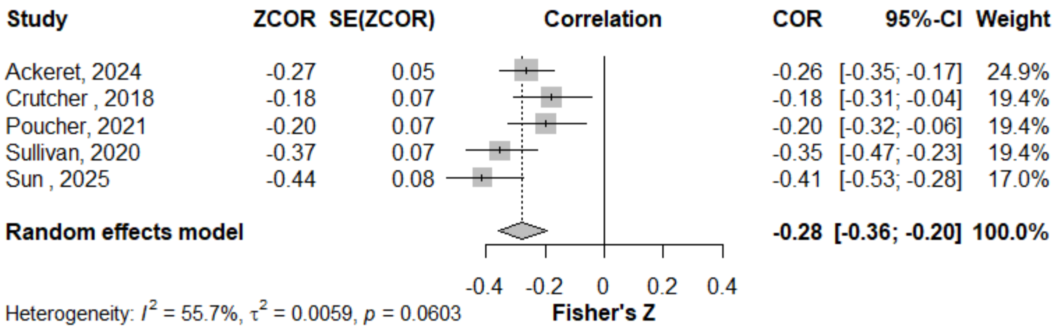

Figure S15. Forest plot of the association between social support and depression

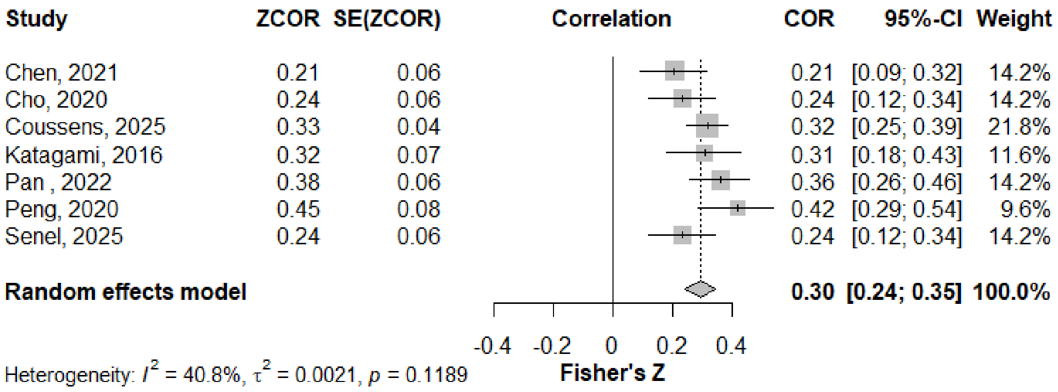

Figure S16. Forest plot of the association between team support and wellbeing

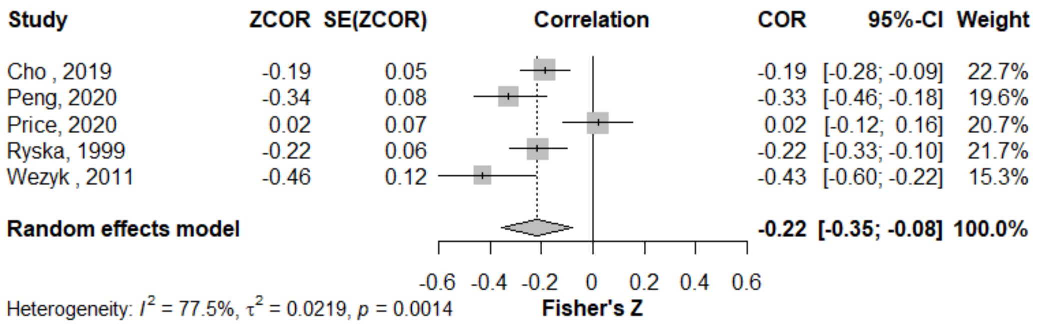

Figure S16. Forest plot of the association between team support and anxiety

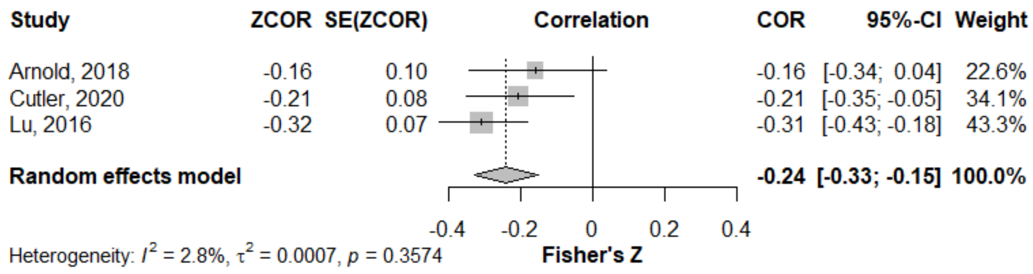

Figure S17. Forest plot of the association between team support and stress

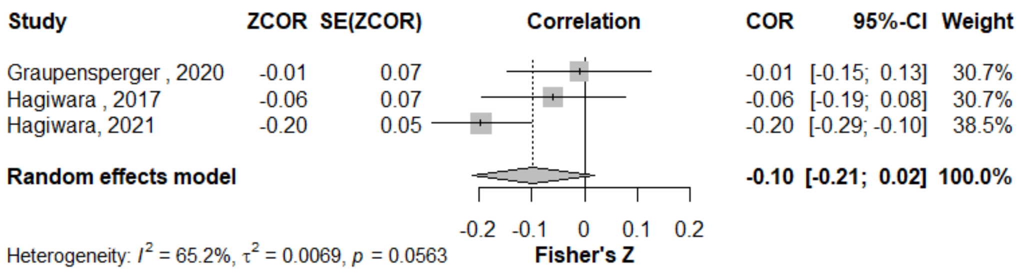

Figure S18. Forest plot of the association between team support and depression

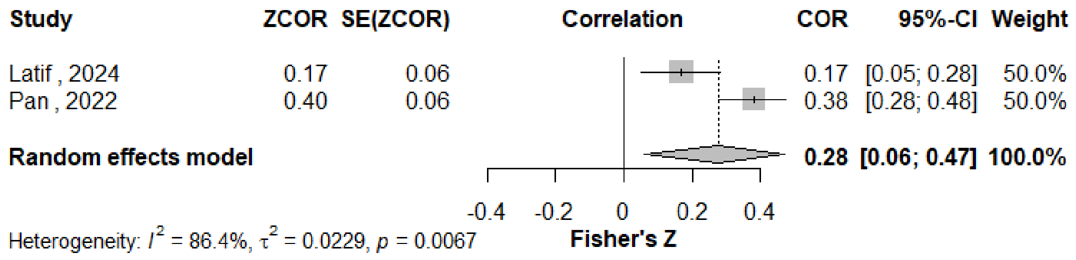

Figure S19. Forest plot of the association between family-friend support and wellbeing

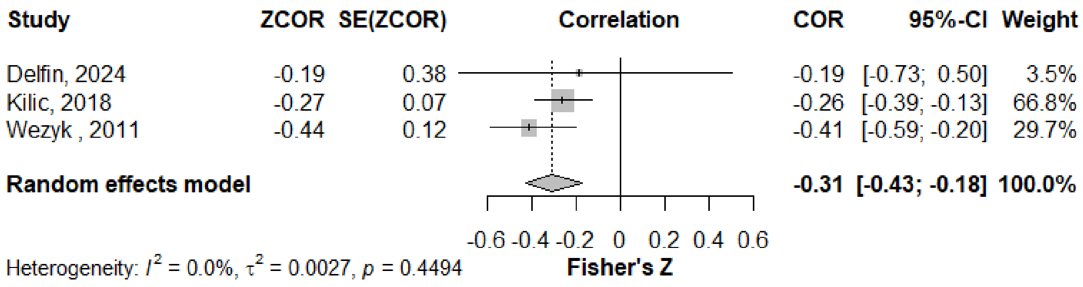

Figure S20. Forest plot of the association between family-friend support and anxiety

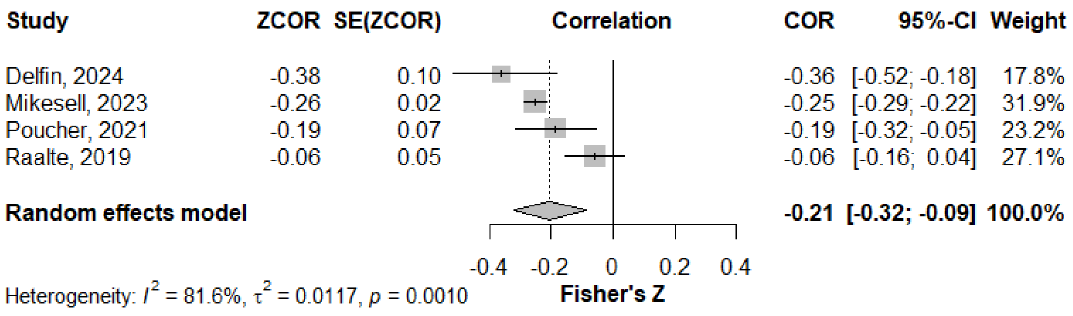

Figure S21. Forest plot of the association between family-friend support and sstress

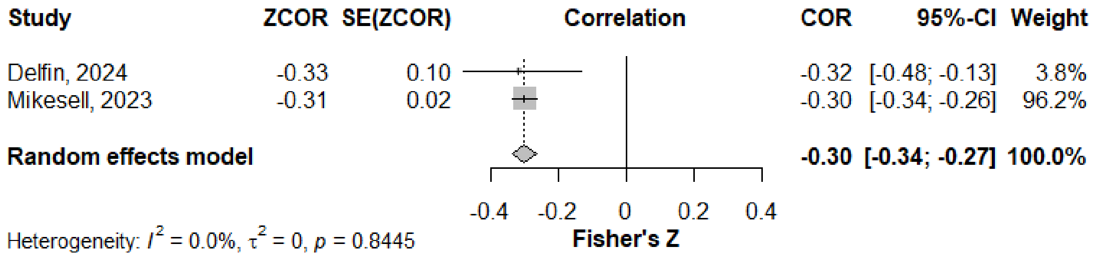

Figure S22. Forest plot of the association between family-friend support and ddepression

S5 File. Moderation Analysis

Table S2. Moderation Analysis of Gender and Athlete Level on the Relationship Between Total Social Support and Mental Health

**Covariate**	***k***	***ES***	**95% CI**	**I^2(%)**	**p-value**
			**Total Wellbeing**		
**Gender (Female%)**					0.827
⩽ 40%	5	0.320	0.245, 0.392	76.2	
40%-60%	4	0.343	0.194, 0.476	85	
≥ 60%	4	0.289	0.177, 0.394	65.9	
**Athlete Level**					0.928
Amateur	5	0.314	0.188, 0.431	80.1	
Professional	8	0.321	0.258, 0.321	73.3	
			**Total Anxiety**		
**Gender (Female%)**					0.233
⩽ 40%	3	-0.315	-0.471, -0.140	64.2	
40%-60%	3	-0.222	-0.300, -0.142	0	
≥ 60%	1	-0.129	-0.261, 0.007	--	
**Athlete Level**					0.122
Amateur	3	-0.185	-0.255, -0.114	0	
Professional	4	-0.287	-0.390, -0.178	39.1	
			**Total Stress**		
**Gender (Female%)**					0.266
⩽ 40%	2	0.300	-0.362, -0.236	0	
40%-60%	3	-0.217	-0.294, -0.138	0	
≥ 60%	3	-0.253	-0.437, -0.048	92.1	
**Athlete Level**					0.816
Amateur	3	-0.246	-0.324, -0.165	16.1	
Professional	5	-0.262	-0.371, -0.147	84.8	

Note: Subgroup analyses were not conducted when fewer than three relatively homogeneous studies were available for each subgroup.

S6 File. Sensitivity analysis

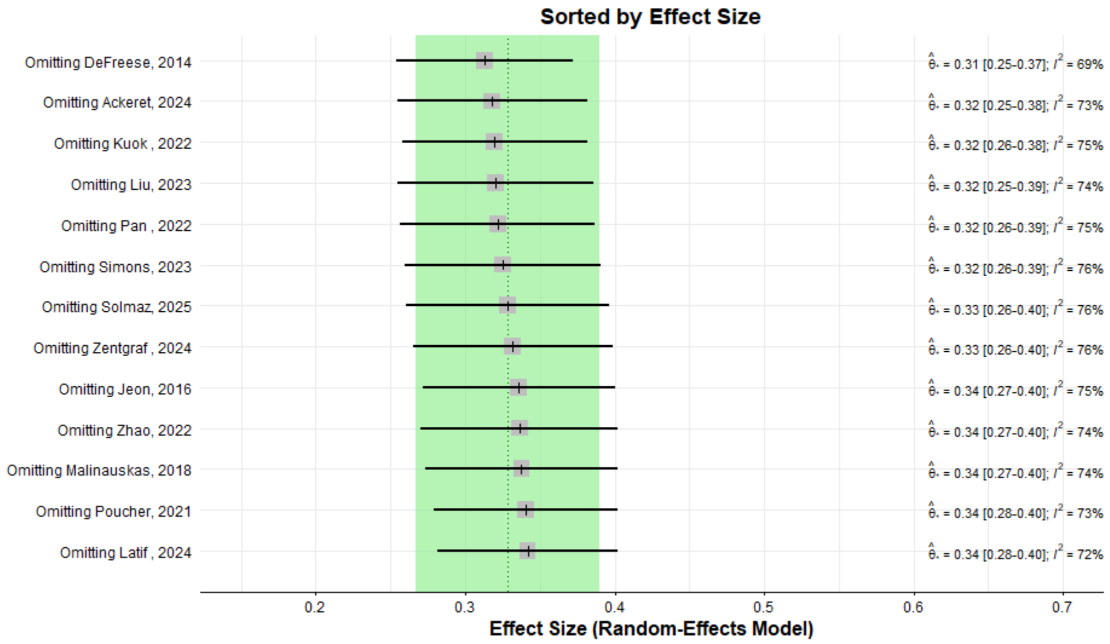

Figure S7. Sensitivity analysis of the association between social support and well-being

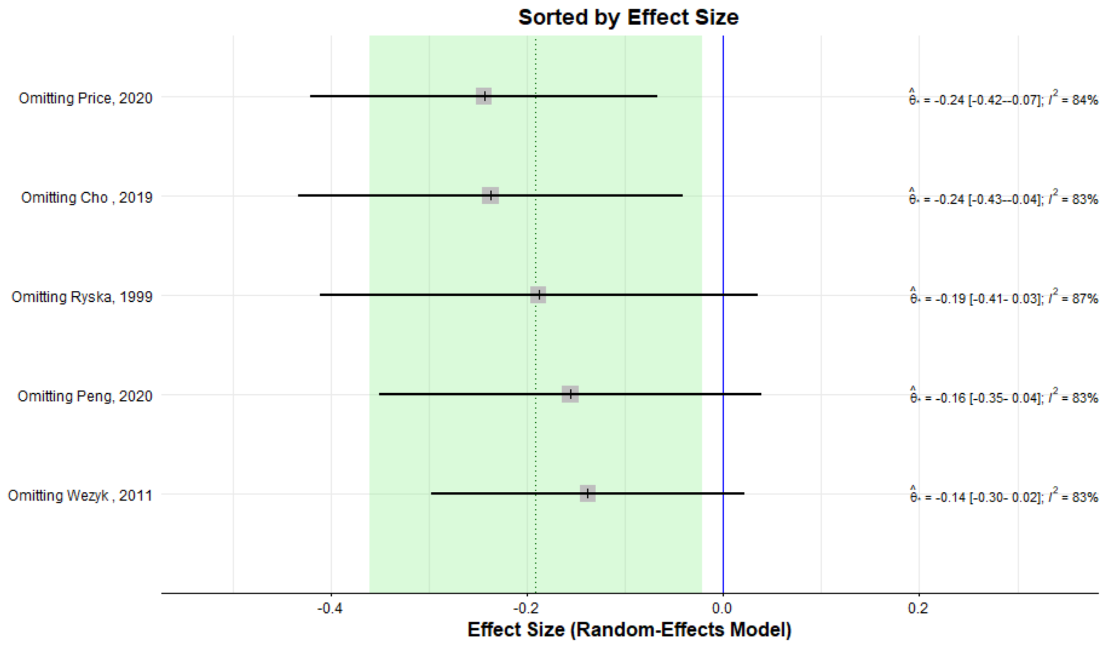

Figure S8. Sensitivity analysis of the association between team support and well-being

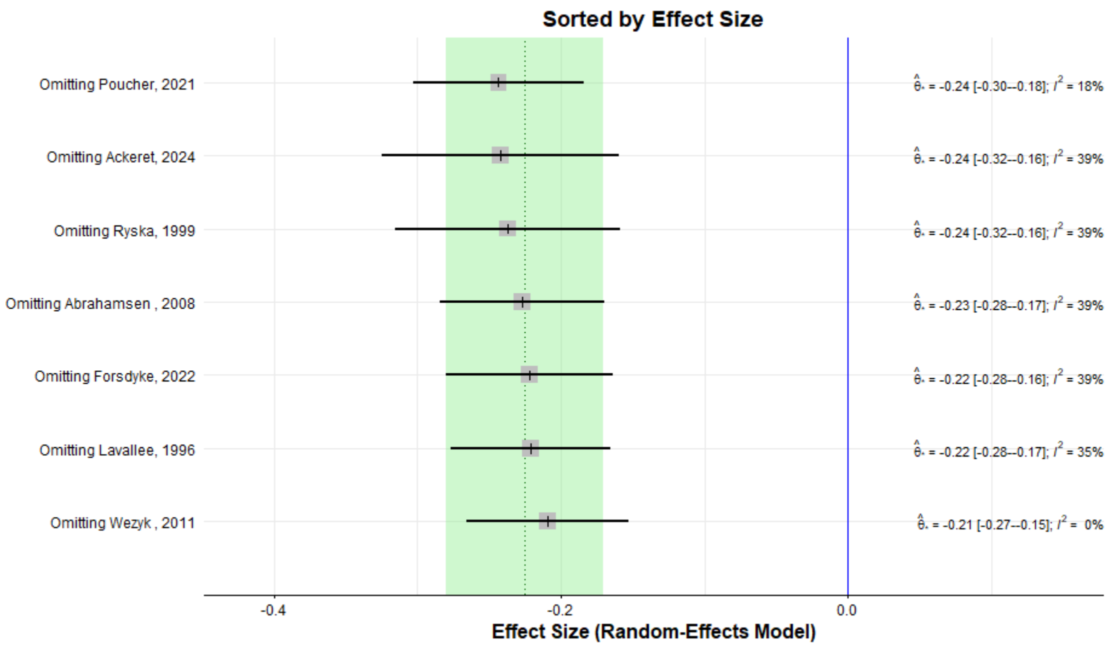

Figure S9. Sensitivity analysis of the association between social support and anxiety

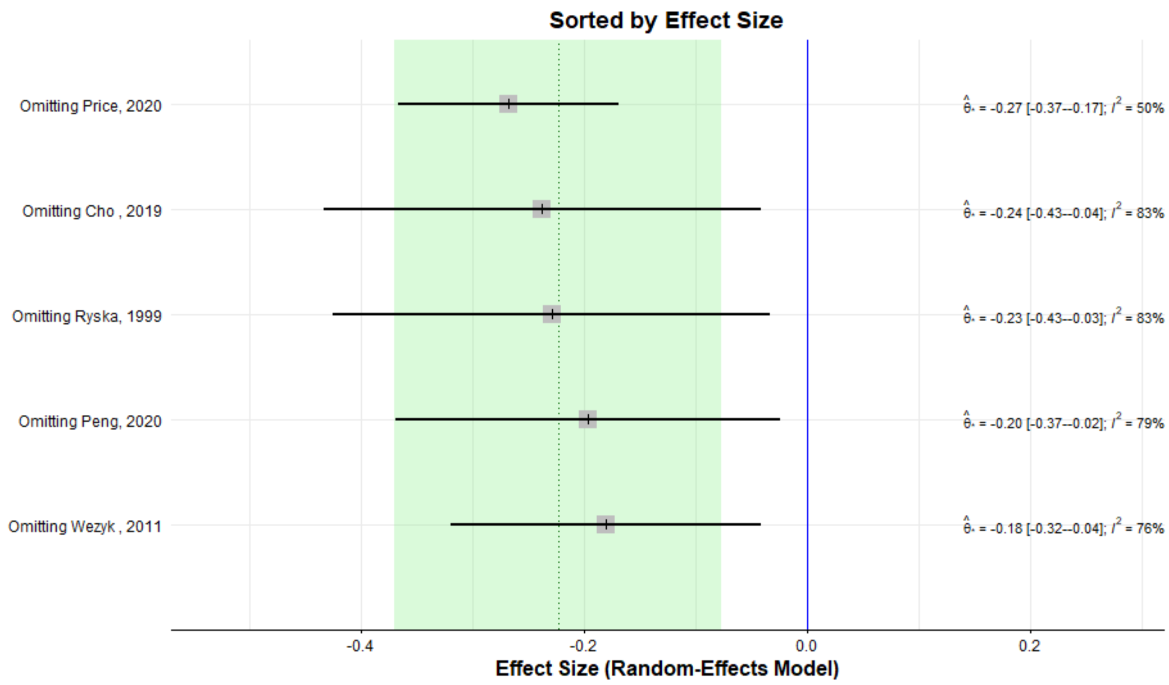

Figure S10. Sensitivity analysis of the association between team support and anxiety

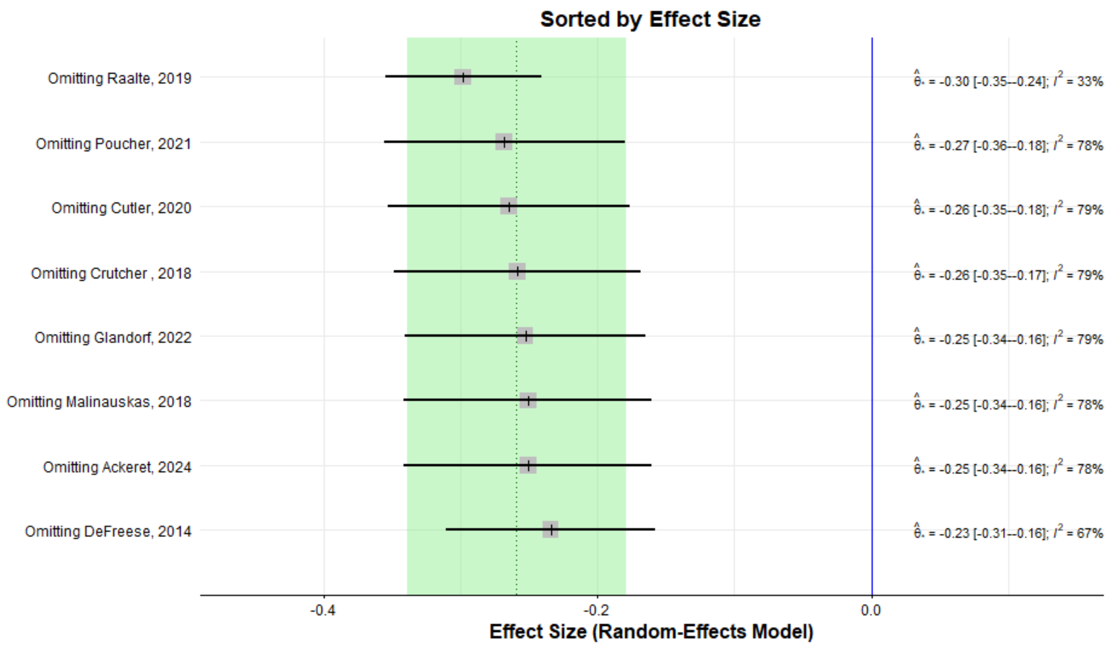

Figure S11. Sensitivity analysis of the association between social support and stress

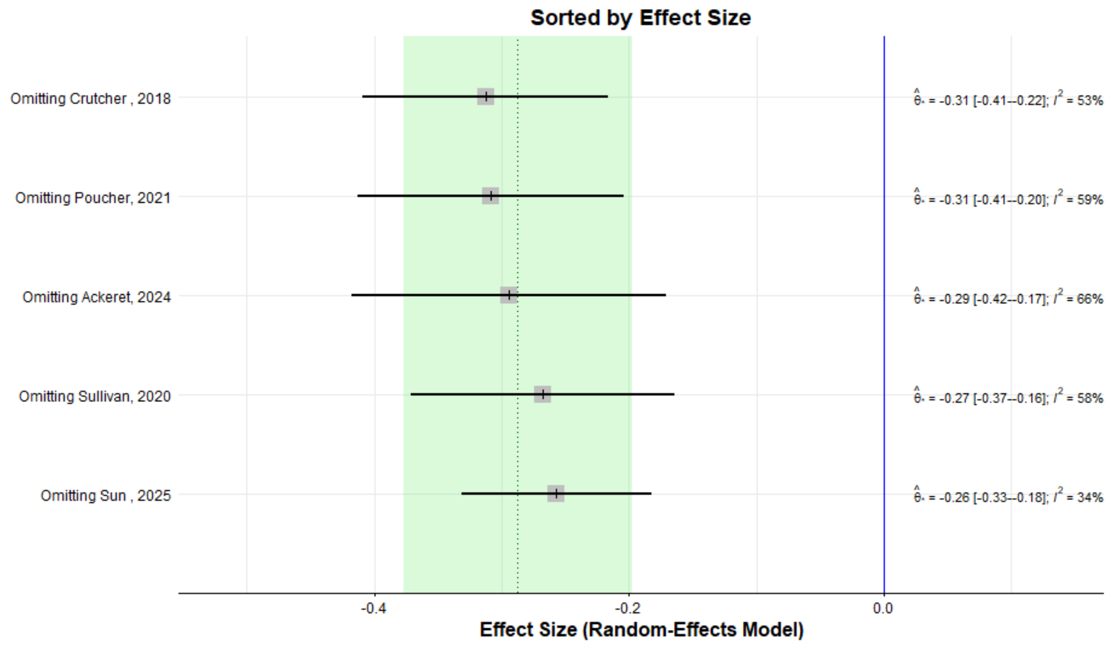

Figure S11. Sensitivity analysis of the association between social support and depression
